# Relationship between thick or greasy tongue-coating microbiota and tongue diagnosis in patients with primary liver cancer

**DOI:** 10.3389/fmicb.2022.903616

**Published:** 2022-12-29

**Authors:** Yuren Zhang, Hetong Zhao, Yuyu Guo, Yongbin Meng, Shasha Yu, Bo Pan, Xiaofeng Zhai

**Affiliations:** ^1^Department of Traditional Chinese Medicine, Changhai Hospital, Naval Military Medical University, Shanghai, China; ^2^Department of Gastroenterology, Shanghai Jiading Hospital of Traditional Chinese Medicine, Shanghai, China

**Keywords:** traditional Chinese medicine (TCM), 16S rRNA gene sequencing, tongue coating microbiota, primary liver cancer, syndrome

## Abstract

Tongue diagnosis is a unique aspect of traditional Chinese medicine for diagnosing diseases before determining proper means of treatment, but it also has the disadvantage of relying on the subjective experience of medical practitioners and lack objective basis. The purpose of this article is to elucidate tongue-coating microbiota and metabolic differences in primary liver cancer (PLC) patients with thick or greasy tongue coatings. Tongue-coating samples were analyzed in 60 PLC patients (30 PLC with thick or greasy tongue-coating patients and 30 PLC with tongue-coating neither thick nor greasy) and 25 healthy controls (HC) using 16S rRNA gene sequencing technology. As compared to healthy individuals, tongue coatings of patients with PLC had elevated levels of *Firmicutes* and *Actinobacteria*. The abundance of *Fusobacteria, SR1_Absconditabacteria_*, and *Spirochaete* were higher in tongue coatings of healthy controls compared to samples in patients with PLC. In addition to site-specific differences, higher abundances of *Fusobacteria* and *Actinobacteria* were observed in thick or greasy tongue-coating patients as compared to non-thick and greasy tongue-coating patients. The inferred metagenomic pathways enriched in the PLC tongue-coating patients were mainly those involved in replication, recombination, and repair of protein. We also identify a tongue-coating microbiome signature to discriminate HC and PLC, including 15 variables on genus level. The prediction performance of the signature showed well in the training and validation cohorts. This research illustrates specific clinical features and bacterial structures in PLC patients with different tongue coatings, which facilitates understanding of the traditional tongue diagnosis.

## Introduction

Primary liver cancer (PLC) is one of the most prevalent malignant tumors and the third leading cause of cancer-related death in the world (Sung et al., 2021), ranks fourth in the incidence of malignant tumors, and third in mortality in China (Chen et al., 2016). Histologically, PLC includes three main subtypes: hepatocellular carcinoma (HCC), intrahepatic cholangiocarcinoma (ICC), and mixed hepatocellular cholangiocarcinoma. Despite recent advances in treatments of PLC, including targeted and immune therapy, the length or quality of life of patients with HCC is still poor. In China, traditional Chinese medicine (TCM) is a wildly used therapy for the prevention and treatment of liver cancer (Xi and Minuk, 2018), and it has contributed to prolonging the survival and improving the quality of life of patients with PLC (Liu et al., 2019).

Tongue diagnosis is a novel diagnostic method in Chinese medicine to determine the health status of patients (Solos and Liang, 2018; Sun et al., 2018). TCM practitioners provide a basis for TCM diagnosis by observing the changes in the characteristics of the dorsum of the tongue, including the shape, size, texture, thickness, color, and so on (Anastasi et al., 2009). It will reveal the functional status of human organs and disease outcomes. Many features are used in the discrimination of TCM Syndromes, but the tongue-coating appearance is one of the major factors. For example, the greasy tongue is an important variable affecting discrimination in Shi-Re (dampness-heat) syndrome. The tongue diagnosis relies on the personal experience of Chinese medicine practitioners and the surrounding environment. However, it lacks certain reproducibility and is difficult to be recognized by the majority of modern doctors (Kamarudin et al., 2017). It is needed to provide a corresponding biological basis for tongue diagnosis in patients.

The oral cavity contains highly diverse microbiota, which includes bacteria, fungi, viruses, protozoa, and archaea. With the in-depth study of microorganisms, the influence of flora on human health and disease has been increasingly concerned by researchers. Many studies have found that the flora is closely related to colorectal cancer (Wong and Yu, 2019), pancreatic cancer (Riquelme et al., 2019), liver disease (Jia et al., 2019; Schwimmer et al., 2019), and other chronic diseases (Nishida et al., 2018; Dabke et al., 2019). The oral microbiome is the second largest microbial system in the human body, smaller than the intestinal microbial system (Human Microbiome Project Consortium, 2012). The oral cavity provides a suitable environment for microbial colonization due to its unique structure (Zhou and Zhang, 2015). The researchers found that it has a complex relationship with oral diseases (Kanasi et al., 2010; Shi et al., 2014) and systemic diseases (Pushalkar et al., 2012; Zhou et al., 2013). Recently, it was suggested that the microbiota dysbiosis of the tongue coat could facilitate the prognosis of several cancers (Ali Mohammed et al., 2021), such as pancreatic head carcinoma (Lu et al., 2019) and gastric cancer (Xu et al., 2021). But, the characteristics of the thick or greasy tongue-coating microbiota in patients with hepatocellular carcinoma are unclear.

Our findings would provide insight into the association between the oral microbiota and PLC, and the distinction between bacterial flora of thick or greasy tongue coating of PLC. It would provide the development of a new precautionary or diagnostic biomarker for patients with PLC.

## Materials and methods

### Study participants

The patients were recruited from the Changhai Hospital, China. From May 2017 to June 2018, after inclusion and exclusion criteria, 60 patients were diagnosed with PLC as well as 25 healthy subjects.

The inclusion criteria were as follows: (1) female or male, more than 18 years old; (2) histopathological or clinical confirmed PLC; (3) the following drugs are not used within 2 months: (a) antibiotics; (b) microbial preparations; and (c) other drugs that affect oral microbes; (4) all eligible individuals voluntarily agreed to participate and gave written informed consent; and (5) history of smoking cessation of more than 1 year. The exclusion criteria were as follows: (1) the healthy control group has a history of the gastric disease; (2) those who have used antibiotics, microbial preparations, or other drugs that affect oral microbes within 2 months; (3) receiving radiotherapy, radiofrequency, and percutaneous ethanol injection within 2 months; (4) serious infection; (5) those who have an oral disease; and (6) those who have other malignant tumors. The experiment was approved by the Ethics Committee of Changhai Hospital, and all participants signed informed consent.

### Sampling collection

All tongue-coating samples were obtained and photographed in the morning before patients' food consumption to avoid the interference of food debris. Two traditional Chinese physicians with more than 20 years of clinical experience diagnosed the type of tongue coatings separately, and the DS01-A tongue diagnostic information acquisition system (DAOSH Co., Shanghai, China) was also used to photograph and analyze the images of the tongue coating. The patients were only recruited when the diagnosis of two physicians and the analysis of DS01-A were consistent. All participants were required to rinse their mouths by gargling sterile saline two times before sampling. Each tongue-coating sample was collected from the middle section of the tongue dorsum using a fresh one-off toothbrush and put into the test tube with saline. The tubes were centrifuged for 10 min at 3,000 r/min, and the precipitates were collected. Samples were stored at – 80°C.

### DNA extraction and PCR amplification

Total DNA was extracted using the E.Z.N.A.^®^ Soil DNA Kit (Omega Bio-tek, Norcross, GA, U.S.) according to the manufacturer's protocols. The integrity of the genomic DNA was assessed by electrophoresis (1% agarose gel). Polymerase chain reaction (PCR) amplification of the V3–V4 region of the bacterial 16S rRNA genes was performed using forward primers 338F (5'-ACTCCTACGGGAGGCAGCAG-3') and 806R (5'-GGACTACHVGGGTWTCTAAT-3'). The PCR reactions were performed in 20 μl PrimerStar HS Premix (AP221-02; TransGen, Beijing, China) that contained 4 μl of 5×FastPfu Buffer, 2 μl of 2.5 mM dNTPs, 0.4 μl of forward primer, 0.4 μl of reverse primer, 0.4 μl of FsatPfu Polymerase, and 10 ng DNA. All samples were amplified on an ABI GeneAmp 9700 (ABI, USA) using the following parameters: 95°C for 2 min, followed by 25 cycles of 95°C for 30 s, 55°C for 30 s, and 72°C for 30 s, and a final extension at 72°C for 5 min. The obtained PCR products were run on 2% agarose gel and purified after size selection repeated three times for each sample and quantified using the QuantiFluor™ ST system.

### Sequence analysis

The PCR product was recovered using a 2% agarose gel, purified using AxyPrep DNA Gel Extraction Kit (Axygen Biosciences, Union City, CA, USA), eluted with Tris-HCl, and detected by 2% agarose electrophoresis. Detection quantification was performed using QuantiFluorTM-ST (Promega, USA). The purified amplified fragment was constructed into a library of PE 2^*^300 according to the standard operating protocol of the Illumina MiSeq platform (Illumina, San Diego, USA) and Shanghai Majorbio Bio-pharm Technology Co., Ltd.

### Bioinformatics analysis

Raw fastq files were demultiplexed, quality-filtered by Trimmomatic, and merged by Fast Length Adjustment of SHort reads (FLASH) v1.2.10 software with the following criteria: (1) The reads were truncated at any site receiving an average quality score of <20 over a 50-bp sliding window; (2) Primers were exactly matched allowing two nucleotide mismatching, and reads containing ambiguous bases were removed; And (3) Sequences whose overlap is longer than 10 bp were merged according to their overlap sequence.

Operational taxonomic units (OTUs) were clustered with a 97% similarity cutoff using UPARSE (version 7.1 http://drive5.com/uparse/), and chimeric sequences were identified and removed using UCHIME. The taxonomy of each 16S rRNA gene sequence was analyzed by the RDP Classifier algorithm (http://rdp.cme.msu.edu/) against the Silva (SSU123) 16S rRNA database using a confidence threshold of 70%.

### Statistical analysis

The alpha diversity, the Relative abundances, and the linear discriminant analysis (LDA) of the effect size (LEfSe) (http://huttenhower.sph.harvard.edu/lefse/) were performed by R software packages. The Wilcoxon rank sum test was used for the differential analysis of continuous variables between the two groups. One-way ANOVA was used to assess the differences between the three groups. The Chi-square test was used for counting data tests. The Chi-square test and *t*-test were used to compare the common characteristics of participants and the non-parametric test was used to compare the indices of alpha diversity and the relative abundances between different types of tongue coating. Correlate analysis was used to establish the commensal relationship. Receiver operating characteristic (ROC) curves were used to analyze the distinguishing ability of the taxa. *P*-value < 0.05 was statistically significant. Statistical analysis was performed using R software 3.6.0.

## Results

### Participant characteristics

**Figure 1:**
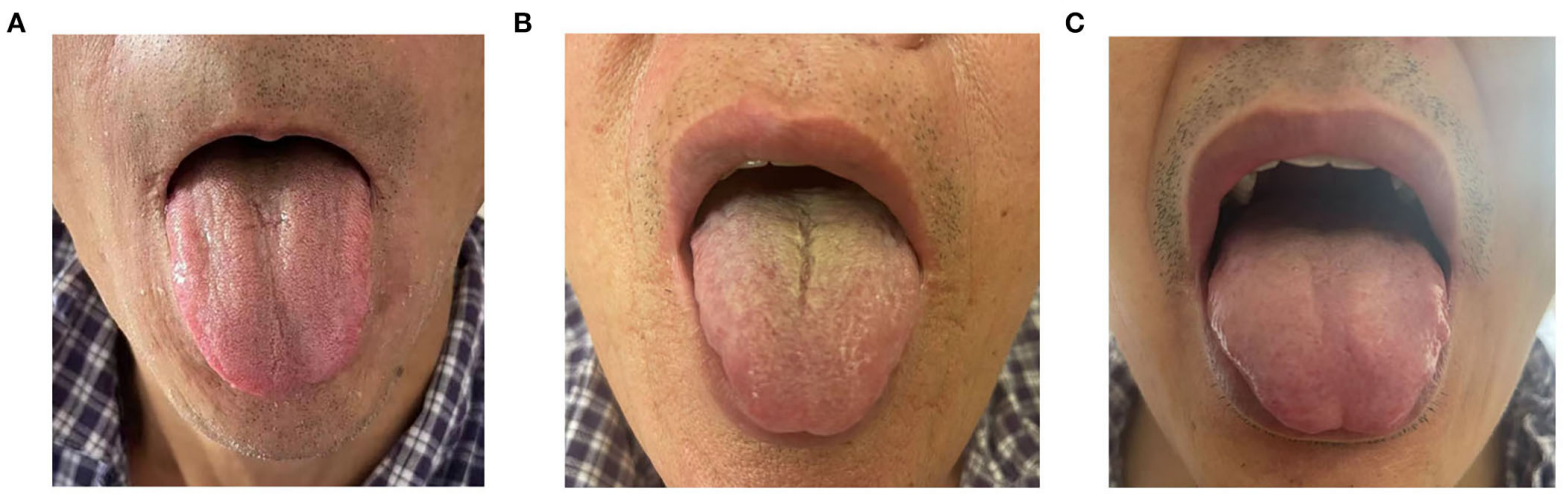
Different tongue-coating images of the three groups. **(A)** Thick or greasy coating (LTG) group (abnormal). **(B)** Non-thick or greasy coating (LNTG) group (abnormal). **(C)** Healthy control (HC) group (normal).

A total of 60 PLC patients and 25 unrelated control subjects were assigned to three groups: (1) PLC with thick or greasy coating (LTG) group (*n* = 30) was comprised of 27 men and 3 women with an average of 57.7 ± 10 years old; (2) PLC with non-thick or greasy coating (LNTG) group (*n* = 30) had 26 men and 4 women with an average of 53.67 ± 11 years old; and (3) The healthy control (HC) group (*n* = 25) had 23 men and 2 women with an average of 56.68 ± 10.359 years (Table 1 and Figure 1). Age, gender, Body mass index (BMI), diabetes, and high blood pressure were comparable between the three groups (*P* > 0.05).

**Table 1:** Demographic characteristics of the three groups.

**Items**	**LTG (*n* = 30)**	**LNTG (*n* = 30)**	**HC (*n* = 25)**	***P*-value**
Gender (male/female)	27/3	26/4	23/2	0.808
Age (Mean ± SD, years)	57.70 ± 10.502	53.67 ± 11.217	56.68 ± 10.359	0.325
BMI (Mean ± SD, kg/m^2^)	23.87 ± 3.037	22.90 ± 3.633	22.48 ± 3.293	0.284
Diabetes	3	2	4	0.529
High blood pressure	4	6	3	0.667

LTG, liver cancer with thick greasy coating group; LNTG, liver cancer with non-thick or greasy coating group; HC, healthy control; SD, standard deviation; BMI, body mass index.

### Tongue-coating microbial dysbiosis

**Figure 2:**
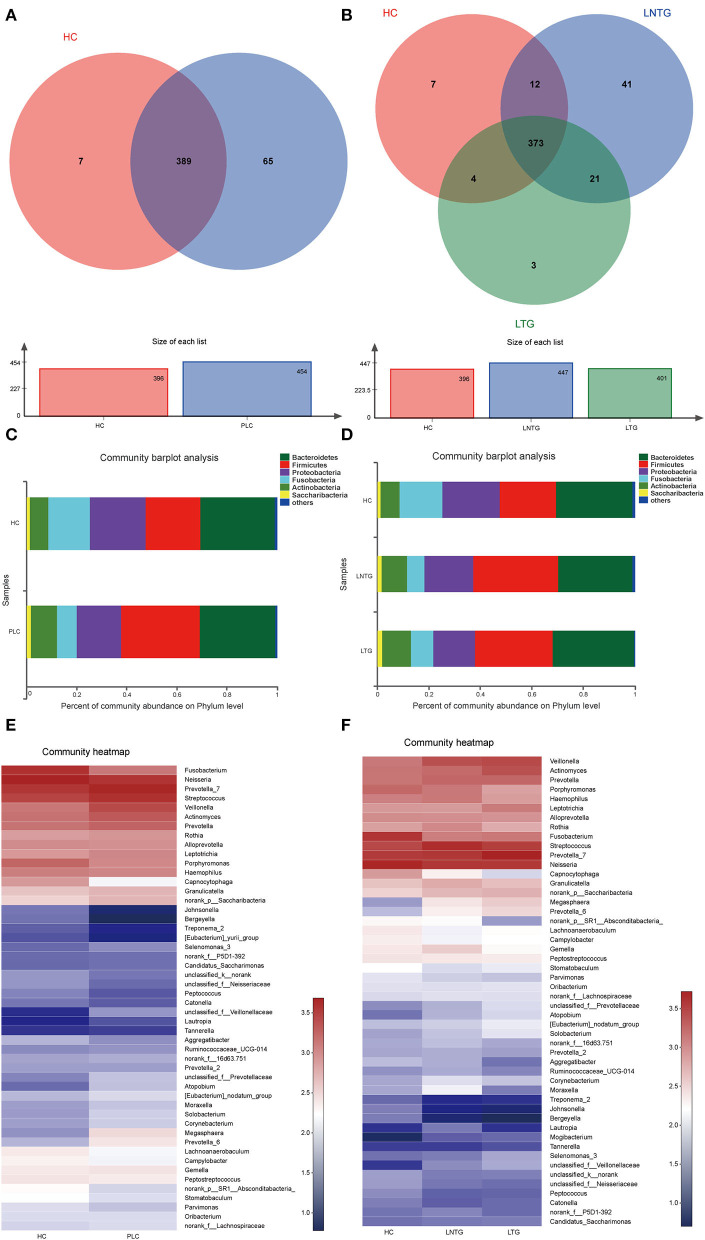
The tongue-coating microbial dysbiosis. **(A)** Venn diagram of the shared OTUs between HC and PLC groups in tongue-coating contents. **(B)** Venn diagram of the shared OTUs among HC, LTG, and LNTG groups in tongue-coating contents. **(C,D)** The relative abundance of tongue-coating microbiota at the phylum level in the two different groups **(C)** and the three different groups **(D)**. **(E,F)** The heatmap of the bacterial community composition at genus level across the two different groups **(E)** and the three different groups **(F)**. (PLC represents primary liver cancer, LTG represents liver cancer with thick or greasy coating, LNTG represents liver cancer with non-thick or greasy coating, and HC represents healthy control).

The Venn diagrams were performed to present the species diversity among the three groups and between the two groups, respectively (Figures 2A,B). There were 389 species in HC and PLC, including 7 species unique to HC and 65 species unique to PLC (Figure 2A). There are 373 species in the three groups; 7 species are not detected in the LTG and LNTG groups, 3 species are unique to the LTG group, and 41 species are unique to the LNTG group (Figure 2B). It was suggested that the species composition among the groups is different and the LNTG group has unique species. Moreover, the species of tongue coating in patients with liver cancer are more than those in the HC group.

The differential abundance of OTUs at the phylum, and genus levels of bacterial taxonomic classification reveal differences in the relative abundance of several taxa among the three groups and between the two groups, respectively (Figures 2C–F). At the phylum level, the community composition of PLC and HC groups includes *Bacteroidetes, Firmicutes*, Proteobacteria, *Fusobacteria, Actinobacteria, Saccharibacteria*, and so on. PLC participants show a slightly higher abundance of *Firmicutes* and *Actinobacteria* and a slightly lower abundance of phyla *Proteobacteria* and *Fusobacteria* (Figure 2C). LTG participants show a slightly higher abundance of *Bacteroidetes, Fusobacteria*, and *Actinobacteria*, and a slightly lower abundance of phyla *Firmicutes* and *Proteobacteria* (Figure 2D). At the genus level, PLC participants showed a slightly higher abundance of *Prevotella_7*, Streptococcus, *Veillonella*, and *Actinomyces*, and a slightly lower abundance of Neisseria and *Fusobacterium* (Figure 2E). LTG participants showed a slightly higher abundance of *Prevotella_7, Actinobacteria*, and *Veillonella* than those of the HC group (Figure 2F). The major bacteria were principally consistent among these groups, but different relative abundances could be observed (Figures 2E,F).

### The tongue-coating microbial diversities

**Figure 3:**
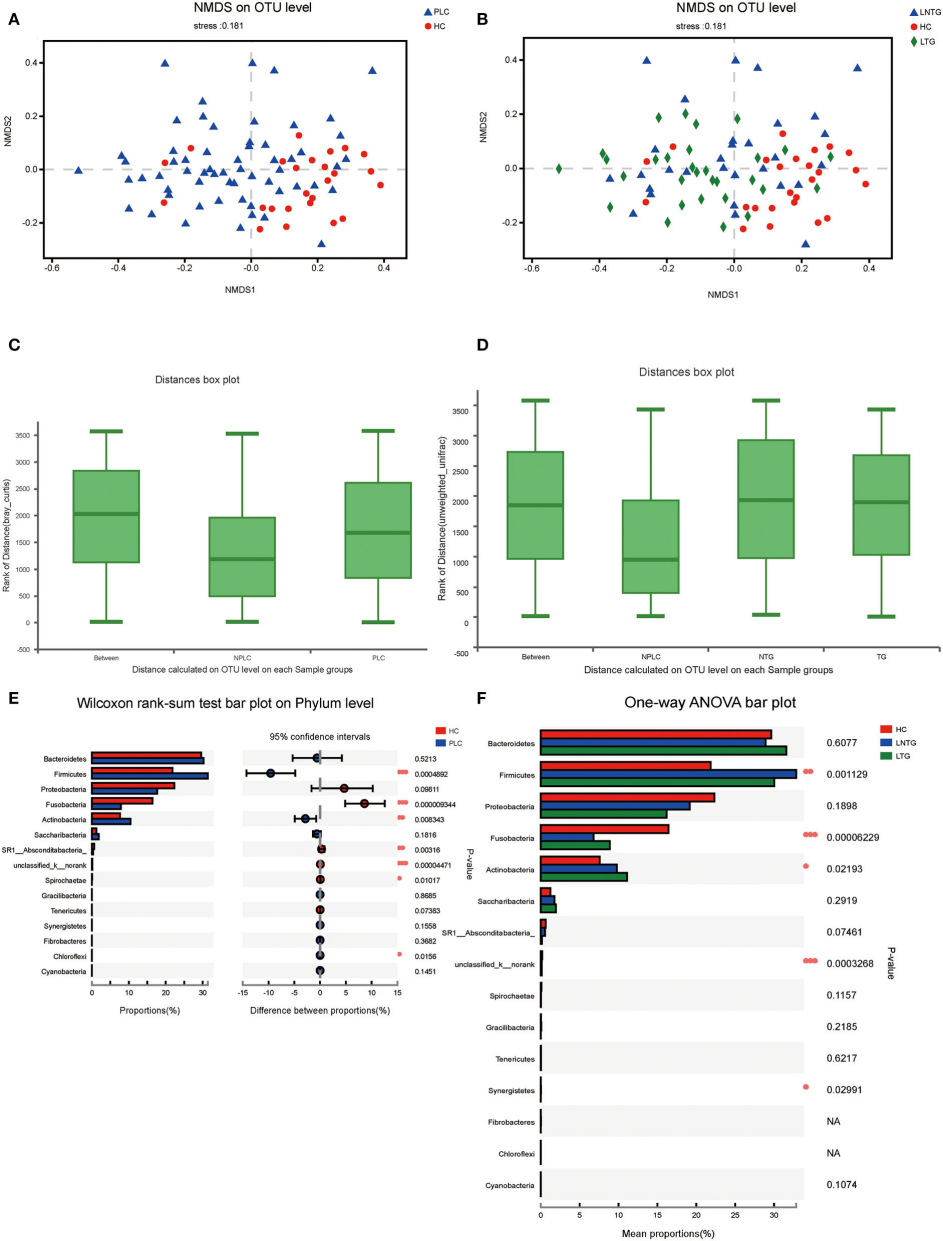
The tongue-coating microbial diversities. **(A,B)** Beta diversity of the tongue-coating microbiota was analyzed using non-metric multidimensional scaling (NMDS) on a Brays–Curtis distance matrix between the two groups **(A)** and among the three groups **(B)**. **(C,D)** Box diagram of the tongue-coating microbiota was analyzed using the ANOSIM test on a Bray–Curtis distance matrix between the two groups **(C)** and among the three groups **(D)**. **(E,F)** Comparison of the microbiome on phylum level of the two groups **(E)** and among the three groups **(F)**.

Similarities of the bacterial communities among the three groups and between the two groups were compared by NMDS and ANOSIM based on Bray–Curtis. NMDS ordination plots of the tongue-coating data showed a clear distinction between the two groups (Figure 3A; NMDS, Stress = 0.181) and among the three groups (Figure 3B; NMDS, Stress = 0.181). ANOSIM revealed significant differences in the structure of tongue-coating microbiota between the two groups (Figure 3C; ANOSIM, *r* = 0.191, *P* < 0.05) and among the three groups (Figure 3D; ANOSIM, *r* = 0.0756, *P* < 0.05). At the phylum level, the Wilcoxon rank-sum test was performed among the three groups and between the two groups. PLC participants showed a higher abundance of *Firmicutes* and *Actinobacteria* and a slightly lower abundance of phylum *Fusobacteria* and *SR1_Absconditabacteria_, Spirochaete* (all *P* < 0.05; Figure 3E). LTG participants show a slightly higher abundance of *Fusobacteria* and *Actinobacteria* and a slightly lower abundance of phylum *Firmicutes* (all *P* < 0.05; Figure 3F).

### Differences in tongue-coating bacterial community composition

**Figure 4:**
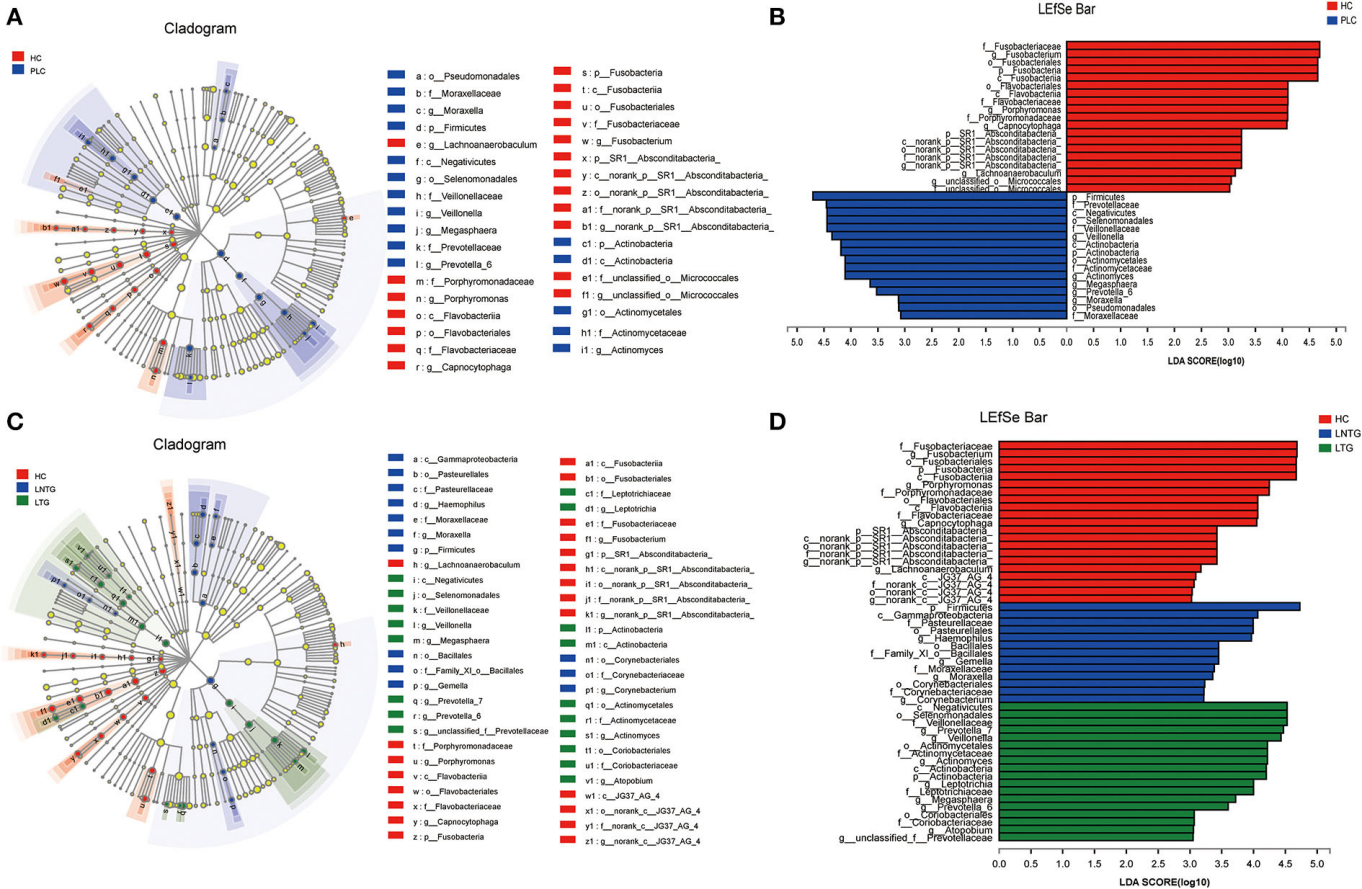
Differentially abundant bacterial taxa in the tongue-coating microbiota were identified by LDA effect size (LEfSe) analysis. **(A,B)** Taxonomic cladogram **(A)** of significant differences and histogram **(B)** of the LDA scores for PLC (blue) and HC (red) groups. **(C,D)** Taxonomic cladogram **(C)** of significant differences and histogram **(D)** of the LDA scores between HC (red), LTG (green), and LNTG (blue) groups. (PLC represents primary liver cancer, LTG represents liver cancer with thick or greasy coating, LNTG represents liver cancer with non-thick or greasy coating, and HC represents healthy control).

The differentially abundant bacterial taxa between the two groups and among the three groups were identified in the tongue-coating microbiota by the linear discriminant analysis effect size (LEfSe) (Figure 4). Between the two groups on genus level, the differentially enriched taxa in PLC groups were mainly from *Firmicutes, Prevotellaceae, c_Negativicutes, o_selenomonadales, Veillonellaceae*, and *Veillonella*, whereas the differentially enriched taxa in HC groups were mainly from *Fusobacteria*ceae, *Fusobacterium, Fusobacteria*les, *Fusobacteria*, and *porphyromonas* (Figures 4A,B). Among the three groups on genus level, the differentially enriched taxa in LNTG groups were mainly from *Firmicutes* and the differentially enriched taxa in LTG groups were mainly from *c_Negativicutes, o_selenomonadales, Veillonellaceae*, and *Prevotella_7* (Figures 4C,D).

### Identification of tongue-coating microbiome signature to discriminate HC and PLC

**Figure 5:**
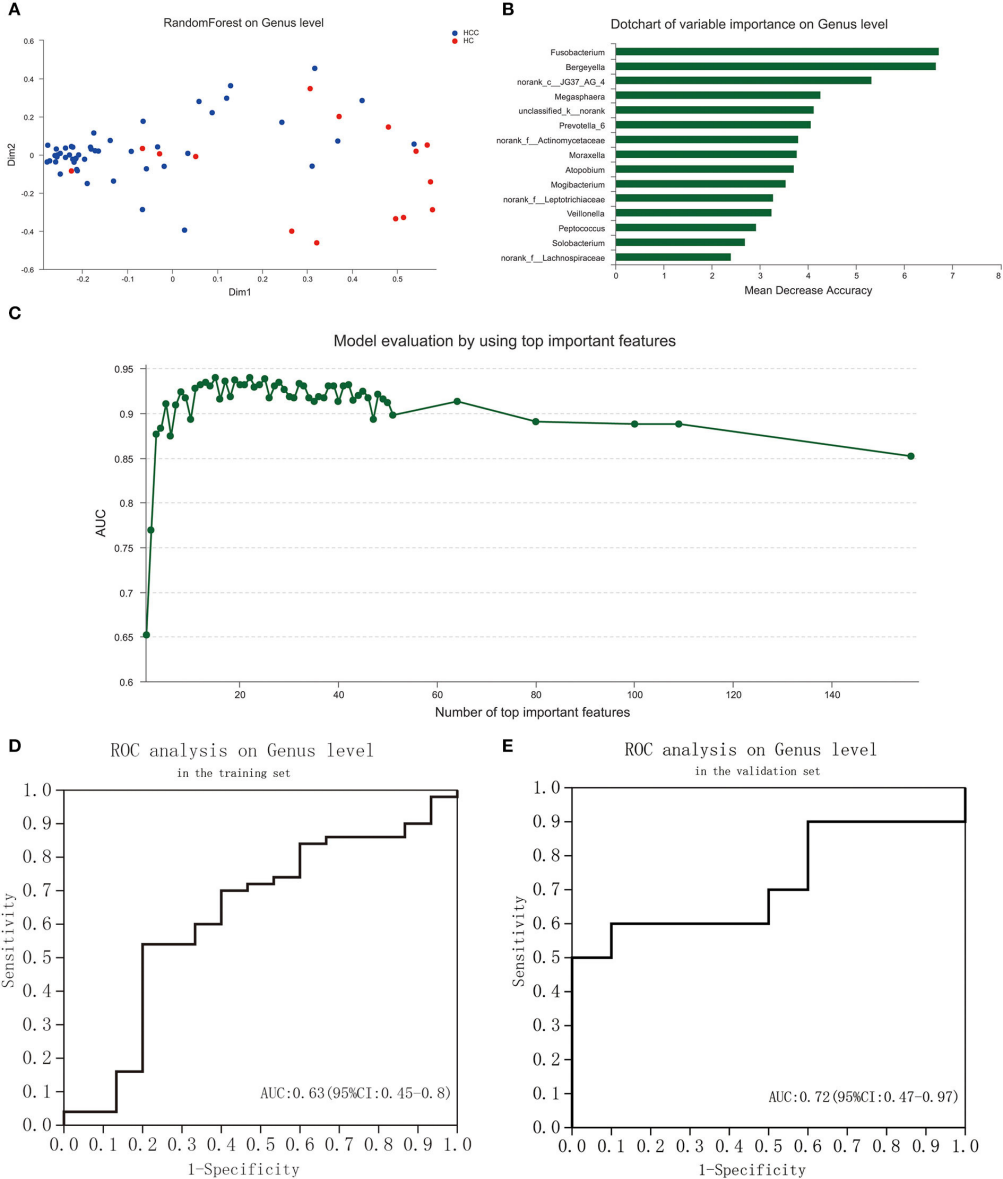
Identification of a tongue-coating microbiome signature to discriminate HC and PLC by random forest analysis. **(A)** The two-dimensional scatter graph represents the similarity of samples in different groups. **(B)** Signature evaluation by using top 10 important features on genus level by random forest analysis. **(C)** Dot chart of top 15 variable importance on genus level. **(D)** ROC of the signature is based on the 15 most important variables in the training set. **(E)** ROC of the signature based on the 15 most important variables in the validation set. (PLC represents primary liver cancer, HC represents healthy control).

To explore the potential ability of the gut microbiome to classify HC and PLC, we constructed a tongue-coating microbiome signature by a random forest model. The two-dimensional scatter graph represents the similarity of samples in different groups (Figure 5A). The number of top important variables in the training set was 15 calculated by a genus-level random forest model with a decision tree for 500 times (Figure 5B). The signature consisted of top 15 variable importance on genus level, including *Fusobacterium, Bergeyella, norank_c__JG37_AG_4, Megasphaera, unclassified_k__norank, Prevotella_6, norank_f__Actinomycetaceae, Moraxella, Atopobium, Mogibacterium, norank_f__Leptotrichiaceae, Veillonella, Peptococcus, Solobacterium, norank_f__Lachnospiraceae*, and *p__Bacteroidetes* (Figure 5C). The area under the ROC curve (AUG) in the training (15 HC and 25 PLC individuals; Figure 5D) and validating (10 HC and 10 PLC individuals; Figure 5E) set were 0.63 (95% confidential interval (CI): 0.45–0.8) and 0.72 (95% CI: 0.47–0.97) based on the 15 most important variables. The 15-microbiome signature showed great diagnostic power to discriminate PLC patients from HC individuals.

### Functional prediction

**Figure 6:**
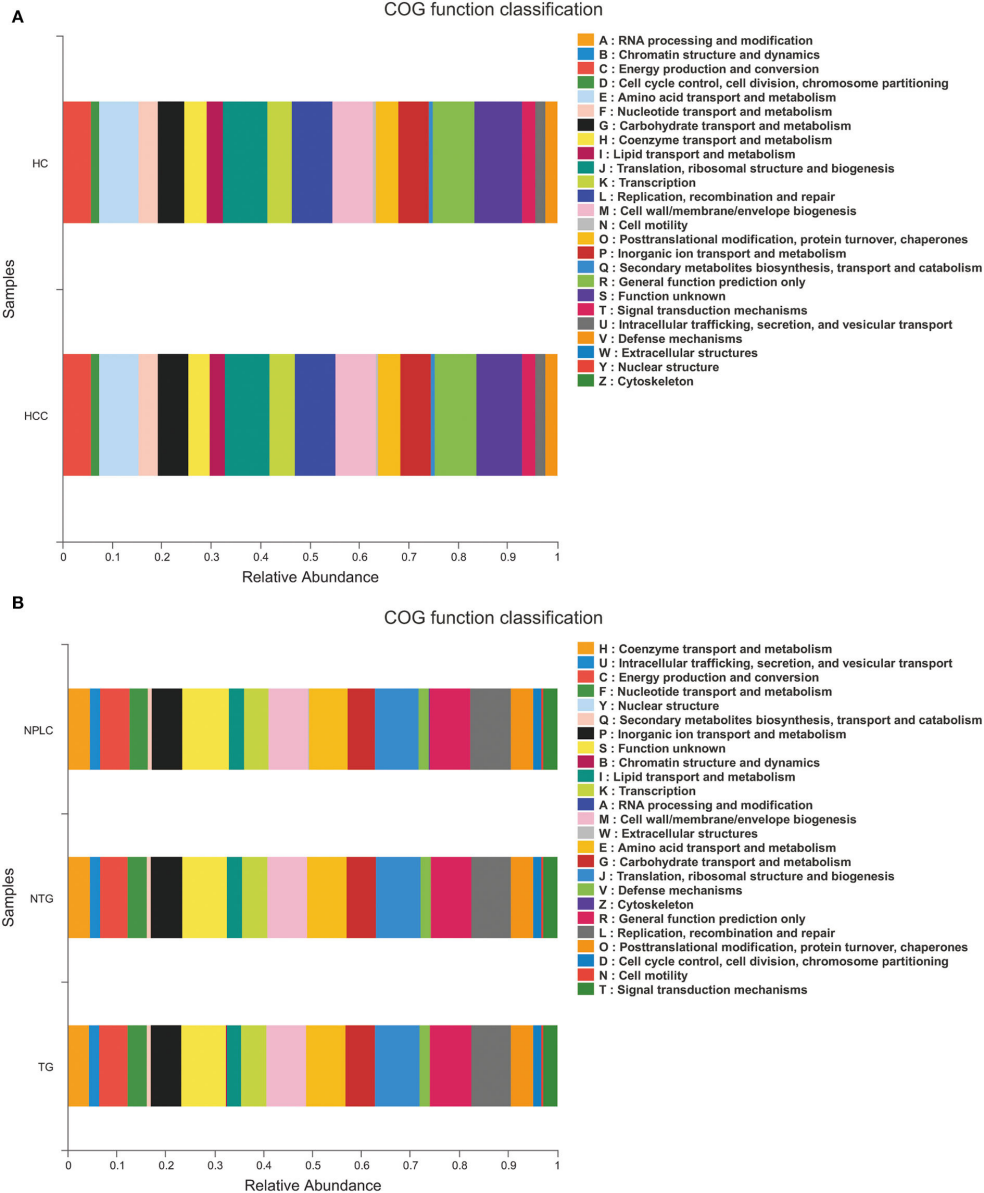
Functional predictions. **(A)** The compositions of COG function in the HC and PLC groups. **(B)** The compositions of COG function in the HC, LTG, and LNTG groups. (PLC represents primary liver cancer, LTG represents liver cancer with thick or greasy coating, LNTG represents liver cancer with non-thick or greasy coating, and HC represents healthy control).

The functional prediction was analyzed to reveal potential function annotation of the Cluster of orthologous groups (COG) family and KEGG Ortholog (KO) using Phylogenetic Investigation of Communities by Reconstruction of Unobserved States (PICRUSt). The top five most abundant functions in HC, LTG, and LNTG groups were: translation, ribosomal structure, and biogenesis; general function prediction; Replication, recombination, and repair; cell wall/membrane/envelope biogenesis; and amino acid transport and metabolism (Figure 6A). The same results of COG function prediction were found between HC and PLC groups (Figure 6B).

## Discussion

Nowadays, PLC still represents a heavy burden for patients and healthcare systems in China, due to high incidence and high mortality. TCM is widely used in all stages of PLC that plays an active role in the prevention and treatment of PLC in China (Hamasaki, 2017; Shi et al., 2017). The TCM Syndrome, known as “Zheng,” is an integral and essential part of TCM theory. It is always used to identify the pattern of patients and guide individualized therapy (Chen and Wang, 2012; Ji et al., 2016). Many features are used in the discrimination of syndromes, but the tongue-coating appearance is one of the major factors. Shi-Re (dampness-heat) syndrome is one of the fundamental syndromes of malignancies and a greasy or thick tongue coating usually indicates dampness-heat syndrome (Sui et al., 2019). In summary, to investigate the underlying mechanisms of greasy or thick tongue coating, we can explore the nature of dampness-heat syndrome and obtain new clues and novel ideas for the objective studies of TCM syndromes.

The main purpose of this part of the study is to try to find evidence of tongue microbiota for greasy or thick tongue coating of PLC and to provide a reference to distinguish PLC from healthy individuals. It was revealed that the relative abundance of *Actinobacteria, Veillonella, Actinomyces*, and *Prevotella_7* in the LTG group was significantly higher than that in HC and LNTG groups. For patients with liver cancer, we found that the relative abundance of *Firmicutes, Actinobacteria, Veillonella*, and *Actinomyces* in the PLC group was significantly higher than that in the HC group. However, Li et al. found that the dominant bacteria on the tongue coating of patients with liver cancer were *Fusobacterium* (Lu et al., 2016), while we found that *Fusobacterium* was highly enriched in patients with HC, which was contrary to this conclusion. We found that *Firmicutes, Actinomyces*, and *Actinomyces* were more abundant in tongue coating in the PLC group, which was consistent with Li et al.'s conclusion. It still needs further in-depth mechanism research to elaborate the results.

Through the discriminant analysis of LEfSe multistage species difference, we found that *Firmicutes, Prevotellaceae, Veillonellaceae*, and *Veillonella* in the PLC group had a great influence on the differential effect. However, *Veillonellaceae* and *Prevotella_7* had a great influence on the differential effect in the LTG group. Our function prediction of tongue coating showed that the functions enriched in the HC group mainly included replication, recombination, and repair, while the function in the PLC group was on the low side. The PLC group is mainly enriched in the pentose phosphate pathway and lysine biosynthesis. It was found that the main function of this group may be to induce the risk of the inflammatory reaction to more inflammatory factors.

Using the 16S rRNA gene, the core function of tongue-coating microbiome could be predicted by the PICRUSt software. Pathways encoding for carbohydrate metabolism and oxidative phosphorylation metabolism were detected. The oral cavity is a major gateway to the human body. Microorganisms colonizing the oral cavity have a significant probability of spreading to the stomach, lung, and intestinal tract. The metagenome prediction validated the role of tongue coating.

Recently, microbiome signatures are presented to discriminate against different diseases, including colorectal cancer (Mangifesta et al., 2018) and gastric cancer (Xu et al., 2019; Wang et al., 2020). These results showed that the identified microbiome signatures could be non-invasive and accurate biomarkers for early diagnosis of cancers. No study to date, however, has investigated such microbiome signature in PLC in the literature. In this study, the microbiome signature to discriminate HC and PLC was identified using the random forest analysis. The signature consisted of a set of 15 bacterial genera capable of PLC with considerably high accuracy. We found that the signatures classified PLC from healthy controls with an area under the curve (AUC) values all >0.6 in the training and validating sets. The results indicate that the constructed signature is powerful to predict the diagnosis of PLC.

There are still some defects in the study. First, the limited sample size of our study might have led to a certain degree of bias in the results. Second, we only analyzed the specificity of tongue-coating microbiome in patients with the damp-heat syndrome of liver cancer and patients with liver cancer, and predict the differences in microbial function by function analysis. Third, we lacked an additional set of tongue-coating microbiome signatures for external validation. There is no further study on how differential microbial communities cause the damp-heat syndrome of liver cancer. High-throughput sequencing is particularly important in the tongue-coating flora.

In summary, we provided biological evidence of tongue diagnosis and dampness-heat syndrome in TCM. We identified a tongue-coating microbiome signature that was capable of accurately distinguishing PLC from healthy controls. The signature showed characteristics of varying abundance, high degree of bacteria interaction, and carcinogenic potentials. It could serve as a potential non-invasive biomarker, which might be suitable for long-term monitoring of PLC.

## Data availability statement

The raw sequence data reported have been deposited in the Genome Sequence Archive (Genomics, Protemics & Bioinformatics 2021) in National Genomics Data Center (Nucleic Acids Res 2022), China National Center for Bioinformation/Beijing Institute of Genomics, Chinese Academy of Sciences (GSA: CRA009244) that are publicly accessible at https://ngdc.cncb.ac.cn/gsa.

## Ethics statement

The studies involving human participants were reviewed and approved by the Ethics Committee of Changhai Hospital. The patients/participants provided their written informed consent to participate in this study.

## Author contributions

YZ and XZ conceived and designed the experiments, edited, and reviewed the manuscript. YG, YM, SY, and BP performed the experiments. HZ wrote the original draft. All authors contributed to the article and approved the submitted version.

## Funding

This work was supported by the Clinical innovation project of Changhai Hospital (2019YXK029).

## Conflict of interest

The authors declare that the research was conducted in the absence of any commercial or financial relationships that could be construed as a potential conflict of interest.

## Publisher's note

All claims expressed in this article are solely those of the authors and do not necessarily represent those of their affiliated organizations, or those of the publisher, the editors and the reviewers. Any product that may be evaluated in this article, or claim that may be made by its manufacturer, is not guaranteed or endorsed by the publisher.
